# Gender Differences in the Impact of a High-Fat, High-Sugar Diet in Skeletal Muscles of Young Female and Male Mice

**DOI:** 10.3390/nu16101467

**Published:** 2024-05-13

**Authors:** Luana Toniolo, Silvia Gazzin, Natalia Rosso, Pablo Giraudi, Deborah Bonazza, Monica Concato, Fabrizio Zanconati, Claudio Tiribelli, Emiliana Giacomello

**Affiliations:** 1 Laboratory of Muscle Biophysics, Department of Biomedical Sciences, University of Padova, 35131 Padova, Italy; 2 Fondazione Italiana Fegato-Onlus, Bldg. Q, AREA Science Park, ss14, Km 163.5, Basovizza, 34149 Trieste, Italy; silvia.gazzin@fegato.it (S.G.); natalia.rosso@fegato.it (N.R.); pablo.giraudi@fegato.it (P.G.); ctliver@fegato.it (C.T.); 3 Department of Medicine, Surgery and Health Sciences, University of Trieste, 34149 Trieste, Italy; deborah.bonazza@asugi.sanita.fvg.it (D.B.); monica.concato@studenti.units.it (M.C.); fabrizio.zanconati@asugi.sanita.fvg.it (F.Z.)

**Keywords:** obesity, Western diet, skeletal muscle, cell metabolism, fiber plasticity

## Abstract

In the context of the increasing number of obese individuals, a major problem is represented by obesity and malnutrition in children. This condition is mainly ascribable to unbalanced diets characterized by high intakes of fat and sugar. Childhood obesity and malnutrition are not only associated with concurrent pathologies but potentially compromise adult life. Considering the strict correlation among systemic metabolism, obesity, and skeletal muscle health, we wanted to study the impact of juvenile malnutrition on the adult skeletal muscle. To this aim, 3-week-old C56BL/6 female and male mice were fed for 20 weeks on a high-fat. high-sugar diet, and their muscles were subjected to a histological evaluation. MyHCs expression, glycogen content, intramyocellular lipids, mitochondrial activity, and capillary density were analyzed on serial sections to obtain the metabolic profile. Our observations indicate that a high-fat, high-sugar diet alters the metabolic profile of skeletal muscles in a sex-dependent way and induces the increase in type II fibers, mitochondrial activity, and lipid content in males, while reducing the capillary density in females. These data highlight the sex-dependent response to nutrition, calling for the development of specific strategies and for a systematic inclusion of female subjects in basic and applied research in this field.

## 1. Introduction

The World Health Organization (WHO) reports that more than 1 billion people worldwide are obese. Among these, 650 million are adults, 340 million are adolescents and 39 million are children (from WHO website, accessed on 15 November 2023, https://www.who.int/news/item/04-03-2022-world-obesity-day-2022-accelerating-action-to-stop-obesity; https://www.who.int/publications/i/item/9789240075634). These data capture general attention because obesity is associated with both short- and long-term comorbidities [1]. Obesity is a complex condition that can be associated with other diseases, including diabetes, metabolic syndrome, and cardiovascular disorders, adversely impacting the quality of life [2]. In children, obesity is not only associated with metabolic impairment and hypertension but also to polycystic ovarian syndrome, obstructive sleep apnea syndrome, and social and psychological problems [1]. These data draw attention to the importance of a proper lifestyle comprising exercise and adequate nutrition starting from an early age. This is because childhood obesity and malnutrition, in addition to concurrent pathologies, may have a fundamental impact on adult health conditions.

Systemic metabolism and the onset of obesity are strictly correlated with skeletal muscle health in a vicious cycle [3]. Skeletal muscle form and function are profoundly affected in obese individuals, resulting in a pathological condition named obese sarcopenia [4,5]. In turn, a sarcopenic muscle has a crucial role in the establishment of fat accumulation and insulin resistance because of the decrease in energy consumption [6,7]. 

Interestingly, while obese sarcopenia was initially mainly described and investigated in adult and aging individuals, more recent studies have also highlighted the presence of this condition in children and adolescents [8,9,10,11]. 

In the already complex vicious cause–effect cycle of obese sarcopenia, biological sex introduces a further variable [12]. In adult individuals, body fat storage differs between males and females [13,14]. Interestingly, post-menopausal women and men accumulate more fat in the intra-abdominal region compared to pre-menopausal women, increasing their risk of developing obesity-dependent disorders [15,16]. In parallel, skeletal muscle form and function is sex- and hormone-dependent [17,18,19], resulting in different metabolic responses to contraction in the two different sexes [20].

Needless to say, obese sarcopenia in adults or in adolescents has different consequences. In light of the long-term impact of adolescent obesity, it is not only important to understand how skeletal muscle contributes to the onset of obesity but it is also central to know if and how its form and function are affected by this condition. 

The principal cause of obesity is a longstanding imbalance between ingested and expended calories, which leads to fat accumulation [21]. One of the major contributors to increased obesity is an unbalanced diet, for example, the so called “Western diet”. The term Western (American) diet refers to a diet characterized by high intakes of fat, carbohydrates, and sugars coming from pre-packaged foods, refined grains, red and processed meat, high-sugar drinks, sweets, fried foods, high-fat dairy products, and many others. More importantly, the Western diet has been linked to a range of chronic diseases, including obesity, type 2 diabetes, cardiovascular disease, and metabolic syndrome [22,23]. 

The literature reports a range of data obtained from experimental models that testify the impact of obesity and obesogenic diets on the skeletal muscles, but these are mainly restricted to high-fat diets, without taking into consideration the impact of sugars, that in the Western diet are ingested mainly via high-sugar drinks and sweets [24]. Aiming at better reproducing the heterogeneity of this diet, recent research envisaged the use of high-fat, high-sugar (HFHS) diets, containing high glucose and fructose [25,26]. Feeding rodents on HFHS diets has been demonstrated to induce obesity and has been exploited to investigate pathologies such as non-alcoholic fatty liver disease non-alcoholic steatohepatitis [25,26], but to date, less information is available on skeletal muscles. In this context, studies on juvenile rats showed that an HFHS diet altered transcriptional patterns and protein profiles [27], impaired gastrocnemius muscle remodeling, reduced the relative force, and induced a shift in the force length relationship, compared to rats fed on a conventional diet [28]. Moreover, a study on mature rats reports loss of the structural integrity of the vastus lateralis muscle and alteration of inflammatory conditions [29].

Aimed at understanding the impact of early-life malnutrition on the histological and metabolic properties of the skeletal muscle in adulthood, we tested the effect of a 20-week HFHS diet in female and male C57BL/6 mice starting at the age of 3 weeks. Our observations suggest that the HFHS diet has a prominent impact on the metabolic properties of adult skeletal muscles and that the alterations are sex-dependent.

## 2. Materials and Methods

### 2.1. Animals and Diet

C57BL/6 mice at the age of 3 weeks were obtained from the Animal House of University of Trieste. Both males and females were used in this study. Animals, individually identified by ear punching, were randomly group-housed in cages in a temperature-controlled environment (22 ± 2 °C) on a 12 h light/dark schedule and fed ad-libitum with a control diet (CTRL, Special Diets Services (SDS) 811,900 VRF1, Witham, UK) or high-fat, high-carbohydrates diet (D12331, Research Diets, New Brunswick, NJ, USA), combined with 42 g/L fructose/sucrose in drinking water, for 20 weeks [25]. The study was approved by the animal care and use committee of the University of Trieste (OPBA: Organismo Per il Benessere Animale) and the competent Italian Ministry (# 56/2022PR). All the procedures were performed according to the Italian Law (D.Lgs.26/2014) and the European Community Directive (2010/63/EU). A maximal effort was made to minimize the number of animals used and their suffering (3R rule). The distress score evaluation was performed throughout the experimental time. The sacrifice was performed by anesthetic overdose (i.p.; ketamine/xylazine, 40 mg/Kg–10 mg/Kg). 

### 2.2. Body Mass Index (BMI) and Homeostasis Model Assessment of Insulin Resistance HOMA-IR (HOMA-IR) Calculation

Intracardiac sampling of the blood (after 6 h fasting), body weight, and naso–anal length measurement were performed after the sacrifice to measure glycaemia and insulinemia and for computation of body composition via BMI.

Glucose was measured in whole blood using One Touch verio IQ1 m (Life Scan Europe, Zug, Switzerland) according to manufacturer’s instructions. Serum insulin content was quantified from the same blood sample by AlphaLISA Insulin Kit (Perkin Elmer, Waltham, MA, USA) following manufacturer’s instructions. HOMA-IR was calculated for each animal according to the following formula: blood glucose (mg/dL) × fasting insulin (μU/mL)/405 [25]. 

### 2.3. Cryostat Sectioning

Soleus muscles were dissected from mice, rinsed in PBS and gently dried, immersed in Tissue-Tek II OCT compound (Sakura Finetek USA, Inc., Torrance, CA, USA), frozen in isopentane cooled in liquid nitrogen, and preserved at −80 °C till sectioning. Then, 10 μm thick transverse serial sections were obtained by using Leica cryostat (CM 1850, Leica Microsystem, Wetzlar, Germany). Sections were airdried and stored at −80 °C till their utilization for histological analyses.

### 2.4. Myosin Heavy Chains Determination and cross-Sectional Area (CSA) Measurement

Air-dried sections were blocked with 5% normal horse serum in PBS, to avoid non-specific binding of the antibodies, and incubated with primary antibodies in a humidified chamber, at room temperature, for 2 h. The primary antibodies utilized were anti-myosin slow, clone NOQ7.5.4D (Sigma Aldrich, Milano, Italy), diluted 1:500; myosin fast, clone MY.32 (Sigma Aldrich, Milano, Italy), diluted 1:1000. Specimens were washed three times with PBS for 10 min and then incubated for 1 h with a horse anti mouse biotinylated antibody (Vector Laboratories, Burlingame, CA, USA), washed three times for 10 min, and incubated with VECTASTAIN ABC Reagent (Vector Laboratories, Burlingame, CA, USA) for 30 min. After washing, the sections were incubated in Diamino Benzidine solution (SigmaFastDAB; Sigma Aldrich, Milano, Italy), until development of staining. Sections were dipped in water, dehydrated with 95% and 100% ethanol, followed by clarification in BioClear (BioOptica, Milano, Italy), and mounted with a resinous medium. Images covering the entire sections were acquired with a Leica DM500 microscope (Leica, Wetzlar, Germany) equipped with a Leica ICC50 digital camera (Leica, Wetzlar, Germany) with a 20× objective. The full section was reconstructed by stitching the images with the Trak2EM function in Fiji software for Mac OS (https://imagej.net/software/fiji/downloads). For fiber type determination, 427 ± 189 fibers were counted per female’s sample and 352 ± 120 per male’s sample. MyHC I fibers were defined as those which had a positive immunoreaction with the antibody against MyHC I (DAB staining) and negative immunoreaction with the antibody against MyHC II, and vice versa for MyHC II fibers. Fibers with a positive immunoreaction for both antibodies were classified as hybrids [30]. The CSA of fibers was determined with Fiji software by carefully drawing a polygon around the perimeter of 50 MyHC I and II fibers.

### 2.5. Periodic Acid-Schiff (PAS) Reaction for Glycogen Detection

Frozen sections were air-dried at room temperature, fixed for 10 min in 4% paraformaldehyde, rinsed in water and immersed in 0.5% Periodic Acid for 10 min, rinsed in running tap water and incubated for 15 min in Schiff reagent (Merck, Darmstadt, Germany). Specimens were washed three times in a sulfurous solution containing 0.5% Potassium Metabisulfite and 0.05 M Hydrochloric Acid, washed for 3 min in tap water, rinsed in distilled water, dehydrated, and mounted with a resinous mounting medium. Images covering the entire section were acquired with a Leica DM500 microscope with a 20× objective, equipped with a Leica ICC50 digital camera (Leica, Wetzlar, Germany), using constant acquisition parameters. The full section was reconstructed by stitching the images with the Trak2EM function in Fiji software and converted to grayscale to measure the intensity of PAS staining. The intensity of staining was evaluated by measuring the mean gray intensity of five 1000 px^2^ fields in each tissue section [31].

### 2.6. Succinate Dehydrogenase Staining (SDH)

SDH staining has been performed as previously described [30,32]. Briefly, frozen sections were air-dried at room temperature for 15 min, transferred to a freshly prepared SDH solution containing Tris-HCl 0.2 M (pH 7.4), Natrium Succinate 0.2 M and Nitro Blue Tetrazolium (Sigma Aldrich, Milano, Italy) 1 mg/mL, and incubated 15–20 min at 37 °C. The enzymatic reaction was stopped by dipping the slides in water and subsequently fixing them for 10 min in 4% paraformaldehyde. Specimens were extensively washed and mounted in gelatin–glycerol. Images were acquired with a Leica DM500 microscope with a 20× objective, equipped with a Leica ICC50 digital camera (Leica, Wetzlar, Germany), using constant acquisition parameters. The full section was reconstructed by stitching the images with the Trak2EM function in Fiji software and converted to grayscale to measure the intensity of SDH staining. The intensity of staining was evaluated by measuring the mean gray intensity of five 1000 px^2^ fields in each tissue section and in a fiber type specific by carefully drawing a polygon around the perimeter of 50 MyHC I and II myofibers.

### 2.7. Sudan Black (SB) Staining

Frozen sections were air-dried and fixed in 10% formalin for 10 min. Sections were then washed 3 times for 1 min in distilled water before being incubated in propylene glycol for 3 min. Sections were then incubated in Sudan Black B solution for 7 min, differentiated in 85% propylene glycol for 3 min, washed 3 times for 1 min in distilled water, and mounted in gelatin–glycerol. Images were acquired with a Leica DM500 microscope with a 20× objective equipped with a Leica ICC50 digital camera (Leica, Wetzlar, Germany), using identical acquisition parameters. The full section was reconstructed by stitching the images with the Trak2EM function in Fiji software and converted to grayscale to measure the intensity of SB staining. The intensity of staining was evaluated by measuring the mean gray intensity of five 1000 px^2^ fields in each tissue section and in a fiber type specific by carefully drawing a polygon around the perimeter of 50 MyHC I and II myofibers.

### 2.8. Alkaline Phosphatase Stain for Capillaries

Frozen sections were air-dried at room temperature for 15 min and transferred to a freshly prepared solution containing 50 mM Tris-HCl (pH 9.5), 0.14 mg/mL Nitro Blue Tetrazolium (Sigma Aldrich, Milano, Italy), 0.07 mg/mL 5-bromo-4-chloro-3-indolylphosphate (BCIP, Sigma Aldrich, Milano, Italy), and 2.3 mM Magnesium Chloride. Sections were extensively washed in water and counterstained in 0.5% eosin, washed in water, dehydrated with 95% and absolute ethanol, cleared in BioClear clearing reagent (BioOptica, Milano, Italy), and mounted with a resinous medium. Images were acquired with a Leica DM500 microscope with a 20× objective equipped with a Leica ICC50 digital camera (Leica, Wetzlar, Germany), using identical parameters. The full section was reconstructed by stitching the images with the Trak2EM function in Fiji software. As previously described [30,32,33], quantitative analysis of capillary density was determined by counting the alkaline phosphatase-positive dots in five 1000 px^2^ fields of each muscle section, and the number of capillaries per fiber was determined by counting alkaline phosphatase positive dots surrounding 50 randomly chosen fibers.

### 2.9. Statistical Analyses

Significant differences among different groups were determined by performing Student’s *t*-test with GraphPad Prism version 10.00, GraphPad Software, La Jolla, CA, USA.

## 3. Results

### 3.1. Body Mass Index (BMI) and Homeostasis Model Assessment Insulin Resistance (HOMA-IR) Index Are Affected by a 20-Week HFHS Diet 

A 20-week HFHS diet induced an increase in BMI and HOMA-IR in both female and male mice (Table 1). Interestingly, the effect of the HFHS diet on these two parameters displayed a higher impact on male mice compared to female mice. The BMI increased about 20% in females and 35% in males, and the HOMA-IR increased about 2 times in females and about 4 times in males. 

### 3.2. Myosin Heavy Chain Expression and Cross-Sectional Area (CSA) Are Differently Altered in Female and Male Mice Fed with an HFHS Diet

**Figure 1:**
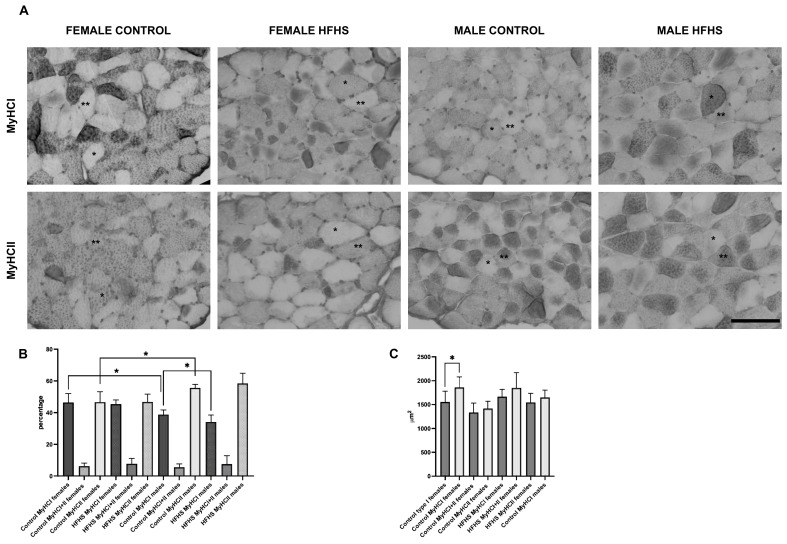
Representative examples of serial sections from soleus muscles sections stained for MyHC I and II (**A**), fiber-type composition (**B**), and fiber CSA (**C**) of female and male C57BL/6 fed on control or HFHS diet. Data on graphs are reported as mean ± SD (* significantly different *p* < 0.05, immumo staining was performed in 4 control females, 10 HFHS females, 5 control males, and 6 HFHS males); one asterisk highlights an MyHC I fiber; two asterisks highlight an MyHC II fiber in the serial sections, Bar 100 μm.

MyHCs expression was assessed by immunohistochemistry reactions. As reported in Figure 1A,B and Figure S1, and the percentage of MyHC I and II was significantly different in female and male control mice (females presented 46.39 ± 2.933 MyHC I fibers, 46.66 ± 6.492 MyHC II fibers and 6.211 ± 1.990 MYHC I and II hybrid fibers; males presented 38.79 ± 2.933 MyHC I fibers, 55.66 ± 2.11 MyHC II fibers and 5.543 ± 2.109 MyHC I and II hybrid fibers), according to previously published data [34]. Interestingly, in HFHS-fed male mice, the percentage of MyHC I fibers significantly decreased from 38.79 ± 2.933 to 34.09 ± 4.31, while did not present statistically significant different values in female mice. In HFHS-fed male mice, the decrease in MyHC I fibers was accompanied by an increase in MyHC I-II hybrid and MyHC II fibers.

As reported in Figure 1C, the measurement of the CSA of MyCH I and MyCH II fibers indicates a general trend to the increase in fiber size. However, only HFHS female mice’s MyHC I fibers displayed a significantly larger CSA. 

### 3.3. Glycogen Content Is Not Affected by HFHS Diet

**Figure 2:**
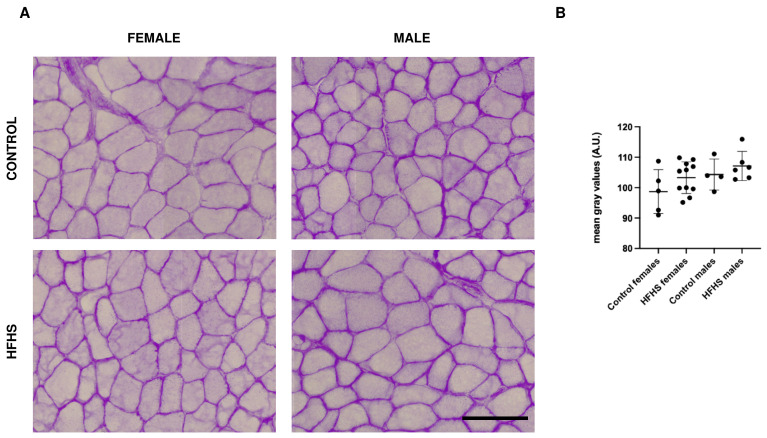
Representative examples of serial sections from soleus muscles sections stained with PAS (**A**) and relative quantification (**B**) of female and male C57BL/6 fed on control or HFHS diet. Data on graph are reported as mean ± SD (PAS staining was performed in 5 control females, 11 HFHS females, 4 control males, and 6 HFHS males); Bar 100 μm, A.U., arbitrary units.

The glycogen content was assessed by means of the periodic Acid–Schiff (PAS) reaction (Figure 2A). As reported in Figure 2A,B and Figure S1, not significant differences were detected between mice fed on a control or an HFHS diet.

### 3.4. Succinate Dehydrogenase (SDH) Assay Reveals an Increase in Mitochondrial Activity in HFHS-Fed Males

**Figure 3:**
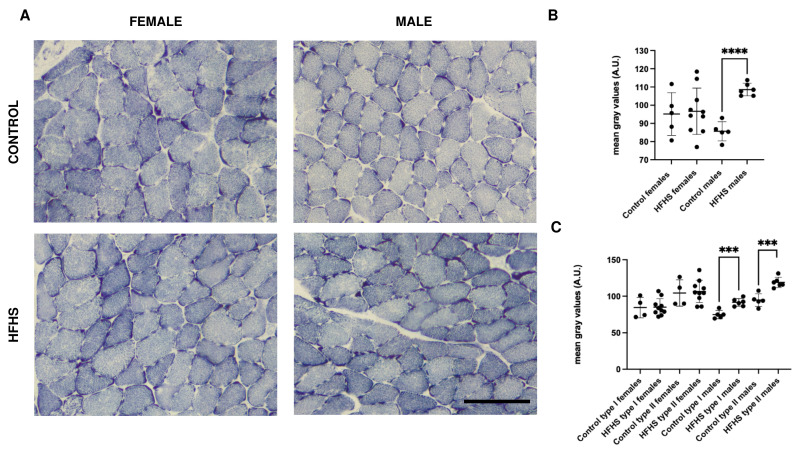
Representative examples of serial sections from soleus muscles sections reporting SDH activity (**A**), global (**B**), and fiber-type specific (**C**) quantification of female and male C57BL/6 fed on control or HFHS diet. Data on graphs are reported with mean ± SD (*** significantly different *p* < 0.001; **** significantly different *p* < 0.0001, SDH staining was performed in 5 control females, 10 HFHS females, 5 control males, and 6 HFHS males); Bar 100 μm, A.U., arbitrary units.

We evaluated the mitochondrial activity by means of the SDH assay (Figure 3A and Figure S1) by initially measuring the intensity of five fields in each section. As reported in Figure 3B, female mice fed on an HFHS diet did not display significant differences compared to controls, while in HFCH male mice sections, the SDH staining was about 20% more intense (*p* < 0.0001). Since in the soleus muscle MyHC II fibers have higher SDH activity, we further investigated whether SDH increase was fiber-type dependent (and, therefore, the increase was mainly dependent on fiber-type switch) or was generalized to all fiber types. As reported in Figure 3C, in males, both MyHC I and II underwent a significant, proportionally equal, increase in SDH activity. It can be concluded that the HFHS diet induces a general increase in SDH activity in male mice, rather than depending on a fiber-type transition. 

### 3.5. Sudan Black Stain Reveals an Increase in Intramyocellular Lipids (IMCLs) in HFHS-Fed Males

**Figure 4:**
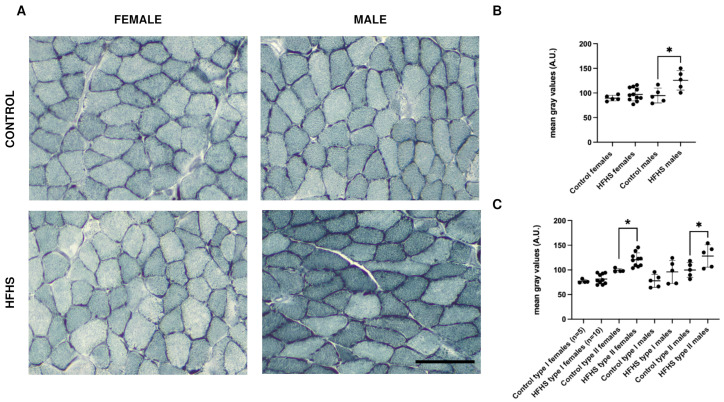
Representative examples of serial sections from soleus muscles sections stained with SB (**A**), global (**B**), and fiber-type specific (**C**) quantification of female and male C57BL/6 fed on control or HFHS diet. Data on graphs are reported with mean ± SD (* significantly different *p* < 0.05, SB staining was performed in 5 control females, 10 HFHS females, 5 control males, and 5 HFHS males); Bar 100 μm, A.U., arbitrary units.

The presence of IMCLs was detected by means of Sudan Black staining. As reported for the SDH staining, we assessed the intensity of staining, both measuring five fields in each section (Figure 4B and Figure S1) and in a fiber-dependent manner (Figure 4C). Interestingly, HFHS-fed females did not present significant differences in the total IMCLs content, while HFHS-fed males accumulated significantly more lipids in the skeletal muscle sections (about +30%, *p* < 0.05). We further investigated whether the SB staining increase was fiber-type dependent or if the increase was correlated to fiber-type switch. As reported in Figure 4C, in female mice, there was a significant increase in IMCLs in type II fibers. In male muscles, both type I and II fibers underwent a comparable increase, but only type II fibers were significantly different. These data suggest that, in these conditions, type II fibers are more prone to accumulate IMCLs. The different significances originating from the global analysis can be explained by the diet-induced increase in type II fibers in male mice. In turn, the differences in fiber-type composition between female and male muscles could serve as a protection from IMCLs accumulation in females.

### 3.6. Skeletal Muscle Capillarization Is Altered in HFHS-Fed Females

**Figure 5:**
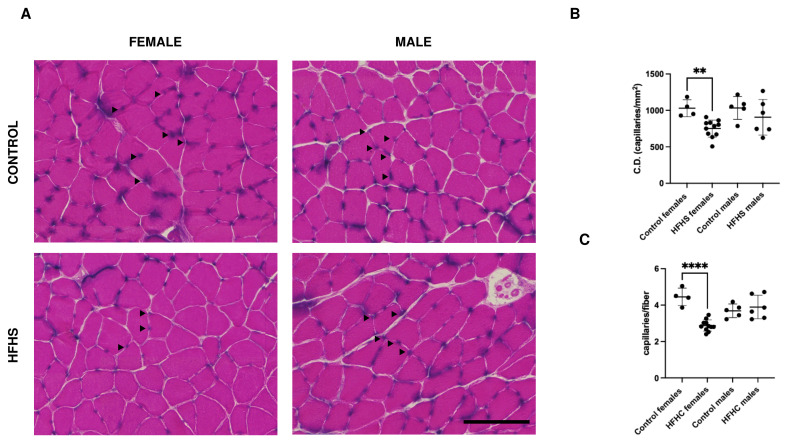
Representative examples of serial sections from soleus muscles sections stained with Alkaline phosphatase to detect capillaries (**A**), and relative quantification of the capillary density (**B**) and capillaries per fiber (**C**) in C57BL/6 female and male mice fed on control or HFHS diet. In panel A, arrowheads point towards some capillaries. Data on graph are reported with mean ± SD; (** significantly different *p* < 0.01, **** significantly different *p* < 0.0001, capillary density and capillaries per fiber have been quantified in 4 control females, 10 HFHS females, 5 control males, and 6 HFHS males); Bar 100 μm, C.D., capillary density.

Capillaries were counted in five fields for each section, and the mean was calculated for each mouse. As reported in Figure 5A,B, capillary density was significantly decreased in females fed on an HFHS diet, while male muscles did not show significant differences. In agreement with this observation, the analysis of the number of capillaries per fiber (Figure 5A,C) showed a significant reduction in the number of capillaries per fiber in HFHS-fed females, while HFHS-fed males did not display significant differences.

## 4. Discussion

The skeletal muscle responds to physiological insults, inducing functional and metabolic modifications. Based on evidence that systemic metabolism, obesity, and skeletal muscle health are strictly correlated, we wanted study the impact of juvenile malnutrition on the adult skeletal muscle by delving into its histo-morphological properties.

In the present work we focused on the soleus muscle of the mouse, which is a slow contracting muscle, responsible for posture and movement. The choice to focus on the soleus was determined by data in the literature reporting that the slow muscles, such as the soleus and diaphragm, are the most affected by high-fat and high-calorie diets [35] and are considered to be similar to human muscles [36]. 

The modification of the MyHCs expression is considered one crucial marker of skeletal muscle adaptation to different physiological changes [37,38,39]. In the mouse, the soleus has been described to present mainly MyHC I (30%) and MyHC IIA fibers (49%), a small percentage of I-IIA (1%) and IIA-IIX (4%) hybrid fibers, and 10% of MyHC IIX fibers [40,41], with some variations depending on the mouse model considered, sex and age, and method of detection. In the present work, we observed that feeding male mice on an HFHS diet induced a significant decrease in the percentage of MyHC I fibers, which was accompanied by a selective increase in MyHC I-II hybrid and MyHC II fibers. These data agree with studies on human muscles, including recent data obtained by Moriggi and collaborators from mice fed on HFDs for 14 weeks and by DeNies and collaborators with a study of a 1-year-long HFD [34]. At variance, female mice did not present statistically different values in fiber composition and only a trend towards an increase in MyHC II fibers was observed. 

The CSA of muscle fibers displayed a general trend towards an increase, but only MyHC I fibers from HFHS-fed females displayed a significantly larger CSA compared lean controls. We think that the difference in the significance between female and male mice depends on the variability of CSA in the male mice fibers, rather than a sex-specific effect of the diet on this parameter. In the mouse soleus muscle, MyHC I fibers have a larger CSA than MyHC II fibers, possibly to maintain the body’s posture. Most probably, the MyHC I-specific CSA increase is the consequence of a higher load during standing, determined by a weight increase. 

Based on evidence that skeletal muscle adaptation involves complex changes in MyHC expressed together with metabolic modulation of the cell [30,42], we delved into the metabolic properties of muscles from HFHS-fed mice, and we found alterations in several parameters.

Interestingly, in HFHS-fed males, the level of SDH activity and the IMCLs were increased in both MyHC I and II fibers. Most probably, the elevated oxidative activity, which in the soleus muscle is possible due to the increase in both MyHC I and IIa fibers, is the response to lipid excess [43]. The condition of high oxidative activity and high IMCLs content could be harmful for the muscle cells. Moreover, the increased mitochondrial respiration could lead to an increase in reactive oxygen species over the physiological levels [44]. A high IMCLs content could compromise excitation–contraction coupling and contractile function of the skeletal muscle [45,46]. Moreover, IMCLs are considered of crucial importance in the development of insulin resistance [47] because they are supposed to interfere with glucose metabolism [48,49]. This is supported by our data showing that female mice, which have a lower SDH activity and a better IMCLs content, have a smaller increase in the HOMA-IR compared to male mice. 

The evidence that IMCLs accumulation is more evident in HFHS-fed male mice could be ascribable to different hormonal status, in agreement with data in humans showing that men and post-menopausal women accumulate more fat in the intra-abdominal region compared to pre-menopausal women, increasing their risk of developing obesity-dependent disorders [16]. According to our observations, in a shorter HFD protocol, Moriggi and collaborators showed that the proteomic profiles of male and female mice were differently affected [50]. Gastrocnemius muscles from male mice presented a slow to fast fiber-type switch, increased oxidative stress, and mitochondrial dysfunction. Contrarywise, the same authors report that females show limited alterations and suggest the concurrent activation of compensatory mechanisms to counteract the increase in fatty acids [50].

Interestingly, our work reveals that capillarization is specifically altered in HFHS-fed females. Considered that MyHC I fibers have a larger CSA, and both capillary density and capillaries per fiber are reduced, it can be suggested that the HFHS diet plays a critical role on muscle physiology by limiting the oxygen and nutrients supply, the removal of waste products, and the regulation of temperature to and from the muscle cell. Interestingly, capillary rarefaction has been reported as an event accompanying obesity [51]. However, in experimental models, this is sometimes controversial [35,52,53]. Although the present analysis does not allow us to suggest a hypothesis, sex [54], age [55], and experimental model [56] could be crucial factors. To our knowledge, different diet-dependent capillary rarefaction has not been described previously and it is difficult to interpret. A possible explanation could be correlated to the lack of induction of the Hypoxia Induced Factor-1α (HIF-1α) in female compared to male mice fed on an HFD [50], interfering with downstream regulators of muscle capillary bed [57]. Messa and collaborators, in an extensive work entailing the analysis of short- and long-term HFD on CD1 female mice in the soleus, extensor digitorum longus, and diaphragm, report a slow to fast fiber-type switch, increased SDH activity, IMCL lipids, and no difference in capillary density in the soleus [35]. The discrepancy with our results could depend on the diet supplied or on the mouse model used. Actually, based on previous work [50] and reported observations, this discrepancy could be due to a different susceptibility of the mice strain to diet, leading us to hypothesize that C57BL/6 females are more resistant (at least for some parameters) to obesity compared to CD1 females, as also described by Messa and collaborators [35]. 

Our evidence confirm that male mice, fed on an HFHS diet in the juvenile age, are more prone to develop obesity and to display alterations to skeletal muscle properties, as demonstrated for other obesogenic diets [34,50]. However, considering the trends towards an increase observed in MyHCs expression, IMCLs content, and SDH activity in female muscles, we cannot exclude that a longer HFHS feeding protocol could also lead to significant differences in female mice. Further studies will be necessary to elucidate this point.

Needless to say, the evidence that the diet has a sex-dependent impact on the skeletal muscle is of fundamental importance for basic research and translational applications. Although recent studies are pointing towards the importance of sex-dependent differences in skeletal muscle form and function [58,59,60,61], very often, research protocols and clinical trials entail mainly males [62]. In this context, in a recent review on human trials to test the effect of Resveratrol on skeletal muscle function, we reported that only 9 out of 24 clinical trials involved women [63]. Based on the distinct sex-dependent development of diet-induced metabolic dysregulation described here and in other works, the potential of skeletal muscle-directed strategies should be reconsidered. For example, altogether, the present histochemical analyses return an overall picture of a muscle that is directed towards an oxidative stress condition, which could entail different mechanisms in the two sexes. It is possible that in males, the increase in mitochondrial activity induces the formation of high amounts of ROS, while in females the alteration of the capillary bed impacts the delivery of oxygen and the scavenging of metabolites and waste products, such as ROS. The different mechanisms of ROS production should be taken into consideration to develop specific strategies. 

## 5. Conclusions

Altogether, the reported data confer to early-life malnutrition a crucial role in the development of sex-dependent metabolic alterations of the adult skeletal muscle. Considering the strict correlation among systemic metabolism and skeletal muscle physiology, these data, once more, indicate this tissue’s target role in the search for strategies to combat obesity. 

Moreover, in the light of the evidence that past experimental works have primarily involved male subjects [64], these data highlight the need to systematically include female subjects in basic and applied research and also in skeletal muscle research. Possibly, this will allow us to obtain important information on sex-specific cellular mechanisms underlying skeletal muscle form and function, and therefore help in the search for specific strategies to contrast skeletal muscle dysfunction. 

## Figures and Tables

**Table 1:** Body weight, length, BMI, fasting glucose, fasting insulin, and HOMA-IR of standard chow (control) and HFHS-fed mice. Data are reported as mean ± SD (significantly different compared to controls * *p* < 0.05, ** *p* < 0.01, *** *p* < 0.0001, **** *p* < 0.0001).

	Control Females (*n* = 5)	HFHS Females (*n* = 11)	Control Males (*n* = 5)	HFHS Males (*n* = 6)
Body weight (mean ± standard deviation)	25.78 ± 1.475	34.82 ± 3.844 ***	31.43 ± 1.279 ****	48.06 ± 3.005
Body length (mean ± standard deviation)	8.72 ± 0.1789	9.145 ± 0.2296 **	9.18 ± 0.2049	9.5 ± 0.2757
BMI (mean ± standard deviation)	34 ± 1	41.64 ± 4456 **	37.60 ± 1.817	50 ± 3.619 ***
Fasting glucose (mean ± standard deviation)	281.6 ± 33.23	293 ± 59.12	255.8 ± 38.28	390.3 ± 71.74 **
Fasting insulin (mean ± standard deviation)	1.150 ± 0.1957	2.356 ± 0.5885 ***	1.524 ± 0.2737	2.716 ± 1.344
HOMA-IR (mean ± standard deviation)	0.8120 ± 0.2239	1.7 ± 0.5287 **	0.9780 ± 0.2905	3.613 ± 2.616 *

## Data Availability

The original contributions presented in the study are included in the article, further inquiries can be directed to the corresponding authors.

## Supplementary Materials

The following supporting information can be downloaded at: https://www.mdpi.com/article/10.3390/nu16101467/s1, Figure S1: Representative examples of serial sections from soleus muscles sections stained for MyHC I, II, PAS, SDH, Sudan Black and Alkaline Phosphatase.
