# Supplementary material for: Gender Differences in the Impact of a High-Fat, High-Sugar Diet in Skeletal Muscles of Young Female and Male Mice
Source: Nutrients. 2024 May 13;16(10):1467. doi: 10.3390/nu16101467 (PMC11124085; doi:10.3390/nu16101467)
Supplement: Supplementary file 1 [file nutrients-16-01467-s001.zip › nutrients-2930008-supplementary.pdf]

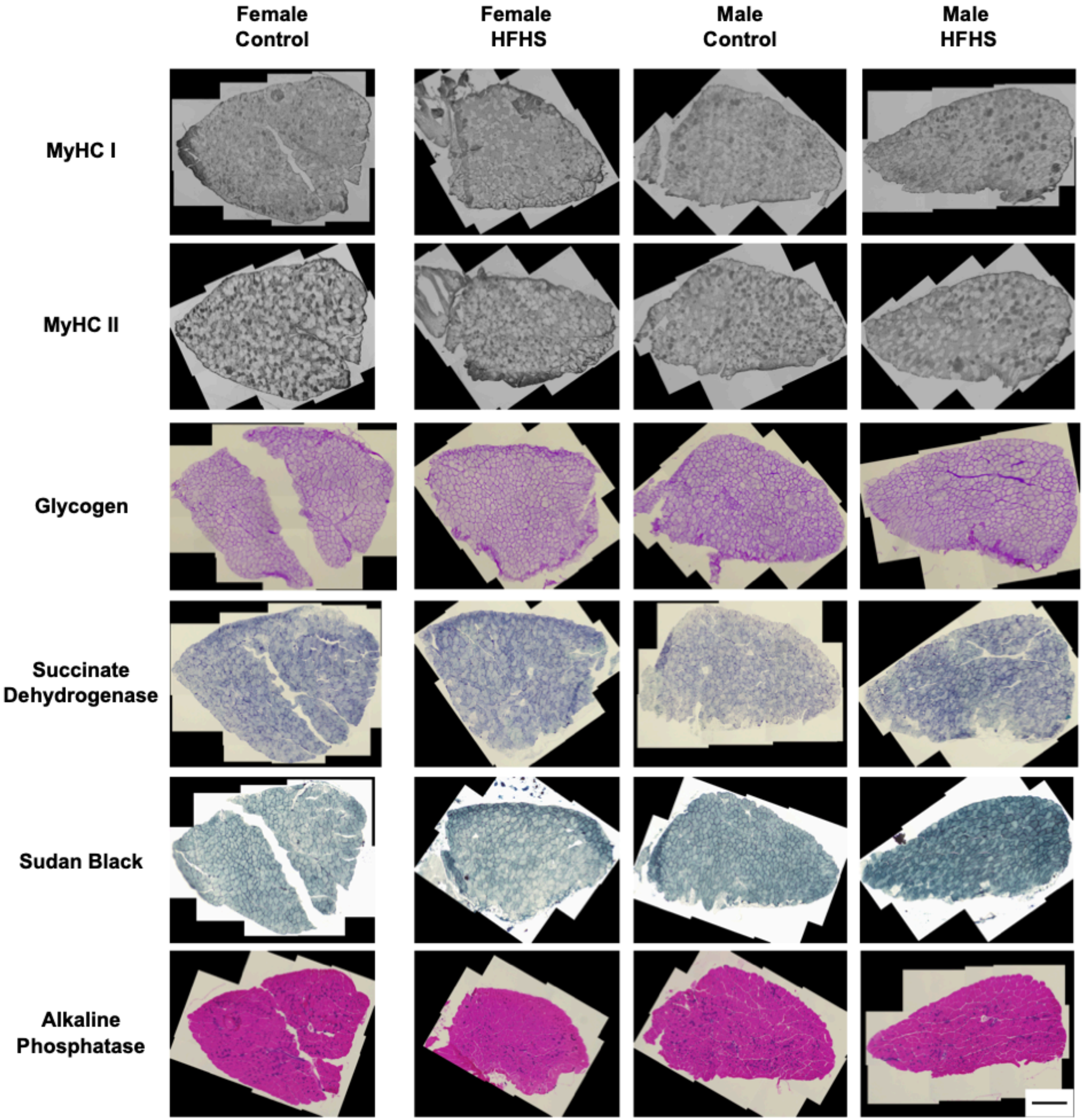

Female Control
Female HFHS
Male Control
Male HFHS
MyHC I
MyHC II
Glycogen
Succinate Dehydrogenase
Sudan Black
Alkaline Phosphatase
